# A two-year review of adult emergency department mortality at Tikur Anbesa specialized tertiary hospital, Addis Ababa, Ethiopia

**DOI:** 10.1186/s12873-021-00429-z

**Published:** 2021-03-19

**Authors:** Hanna Daniel Yosha, Achamyelesh Tadele, Sisay Teklu, Kidest Getu Melese

**Affiliations:** 1 Department of Emergency Medicine, School of Nursing, Addis Ababa University, Addis Ababa, Ethiopia; 2 Department of Obstetrics and Gynecology and Emergency Medicine, School of Medicine, Addis Ababa University, Addis Ababa, Ethiopia; 3 School of Midwifery, College of Health Science and Medicine, Wolaita Sodo University, Wolaita Sodo, Ethiopia

**Keywords:** Early mortality, Emergency department, Ethiopia, Tikur Anbesa tertiary specialized hospital

## Abstract

**Background:**

Adult emergency department mortality remains high in resource-limited lower-income countries. The majority of deaths occur within the first 24 h of presentation to the emergency department. Many of these mortality’s can be alleviated with appropriate interventions. This study was aimed to assess the magnitude, cause, and factors related to very early mortality in patients presented to the emergency department of Tikur Anbesa Specialized Tertiary Hospital, Ethiopia from March 2018 to 2020.

**Methods:**

This is a cross-sectional retrospective chart review. Retrospective data were collected from the records of all patients who died within 72 h of emergency department presentation from March 2018 to 2020. Data entered using Epi data 4.2.1 and analyzed using SPSS Version 23. Using the Chi-square test, binary and multiple logistic regression analysis were carried out to measure the association of variables of interest and very early emergency mortality. *P*-value < 0.05, odds ratio with 95% CI were used to identify the significant factors.

**Results:**

Between March 2018 to 2020, 30,086 patients visited the ED and 604 patients died within 72 h of presentation (274 died within 24 h and 232 within > 24–72 h). Shock (36.7%) and road traffic accidents (3.16%) were the major causes of death. Triage category red AOR 0.23 95% CI 0.1–0.55 and duration of illness 4–24 h AOR 0.47 95% CI 0.26–0.87 were significantly associated with decreased very early emergency department mortality. Meanwhile, co-morbid disease HIV AIDS AOR 2.72 95% CI 1.01–7.30 and residence Addis Ababa AOR 2.78 95% CI 1.36–5.68 and Oromia AOR 3.23 95% CI 1.58–6.54 were found significantly associated with increased very early emergency department mortality.

**Conclusions and recommendations:**

The mortality burden of a road traffic accident and shock in the TASTH is significant and the magnitude of ED mortality differs between these groups. Residence Addis Ababa and Oromia, triage category red, co-morbid disease HIV AIDS, and duration of symptom 4–24 h were significantly associated with early emergency department mortality. Early detection and intervention are required to minimize emergency mortality.

## Introduction

Emergency Department (ED) is a multifunctional unit at which patients are guaranteed access to 24/7 emergency health care service at any level of a health facility. The department is the backbone of the health facilities and the general public by providing the first line of care on arrival. In short, ED is the “shop window” of the health service [1].

According to the Ethiopian Hospital Services Transformation Guideline, the maximum duration of stay allowed in ED is 24 h, however, due to lack of a bed, delay of investigations, and more reasons, patients may stay above 24 h in ED. However, it is expected that all ED care will end with a maximum period of 72 h of the duration of stay [2]. The description of early ED mortality is significant as early mortality is a deputy for disease severity, and it identifies a subgroup of clients who are more likely to benefit from early ED interventions. Early ED mortality is defined in this study as death within 72 h of ED presentation, and very early ED mortality is defined as death within 24 h. Very early mortality group characterizes those patients who are severely ill and are more likely to benefit from ED timely interventions [3, 4].

Emergency department mortality has a profound impact on the individual, society, and the entire health system. It is becoming one of the leading causes of death in hospitals. Globally, it contributes to 15–16% of all mortalities in hospitals. The value is much higher in Low-and Middle-Income Countries (LMICs). In Sub-Saharan Africa, especially in central, east, and west, it is 5.1% higher than in higher-income countries [5, 6].

In recent years, a significant proportion of the burden of disease and patient mortality isaggravated in ED [7, 8]. The major causes of this mortality are cardiovascular disease, traffic accidents, trauma, and cancer. Even if, the etiologies differ in various geographical locations, it has been reported that these causes cover 15–60% of all the mortalities [9, 10]. Decades of advances in clinical science and care delivery have dramatically improved patient outcomes for a range of acute conditions, in higher-income countries. Clinical interventions like standardized ED trauma protocols have been previously shown to decrease mortality in high and few middle-income countries [10]. Moreover, the severity of injury or illness can be the cause of death on its own. The care and management at the hospital also determine the fate of the patients’ life or death, so do lack of, or inadequacy of medical installation and the staffs, thereof inaccessibility of transportation due to the topography of patients’ residency and unsuitable (or unavailable) road network delays arrival of the patients to the hospital, lack of rapid transfers of the patient to other departments (for definitive treatment) which aggravate illnesses or injuries and could cause death also increases the ED burden. These and other pre-hospital factors, like adequate trained man-power, have a direct relationship with the morbidity and the mortality pattern in ED [11, 12].

Ethiopian Hospitals ED mortality from traumatic and medical causes remains largely uncharacterized. Causes are believed to be multifactorial and include both a significant burden of trauma and diseases as well as limited access to quality resources including lack of skilled health care providers [3, 13, 14]. Even though the problem is a huge burden for the community, the health system, and the country in general, evidence in low-income countries specifically in Ethiopia lacks adequate data and this has made it difficult for policy-makers to make major new investments in emergency care. Therefore, this study aimed to determine the magnitude of early emergency department mortality, to characterize all causes of early mortality, and to identify associated factors of very early ED mortality among patients visiting the emergency departments of Tikur Anbesa Specialized Tertiary Hospital (TASTH), Ethiopia from March 2018 to 2020.

## Methods

### Study design and period

A retrospective card review of cases presented to TASTH ED from March 2018 to 2020.

### Study setting

Tikur Anbesa Specialized Tertiary Hospital is found in Addis Ababa, Ethiopia. The hospital has 800 beds, with more than 130 specialists and 50 nonteaching doctors. It offers diagnostic testing and treatment for approximately 370,000–400,000 patients per year. The emergency department of TASTH has 9 consultants and 28 residents and serves 18,000 patients per year starting from age 13. On average, 50 persons that encompass traumatized and/or critically ill patients are seen in the ED per day and many of them require emergency care or resuscitation [15].

### Inclusion and exclusion criteria

Study data include patients who came to ED of TASTH during the study period and dead within 72 h of presentation. Patients who died on arrival and those with an incomplete chart record were excluded.

### Data and materials

Early ED mortality was defined as death within 72 h of ED presentation [3]. A standardized pre-tested data collection form that was developed based on previous literatures [3, 14, 16–18] was used to assess the study objectives. Raw data on adult emergency mortality were retrieved from a secondary data source which includes a Health Management Information System registration book, patient chart, ED triage record, clinical care note, and hospital death certificate. Trained nurse professionals participated in assisting the data collection process. The questioner encompasses socio-demographic characteristics, clinical data on mode of transportation to ED, source of referral, duration of symptom, clinical and diagnostic factors, the medical and nonmedical cause of death, and triage category.

Age categorized into i. 13–24 ii. 25–53 iii. 54–64 iv. > 65 [19]. Clinical data includes mode of transportation, prior ED visits in the past 30 days, duration of symptoms, length of stay in the emergency department, time of death, source of referral was categorized to i. Private hospital ii. Public hospital iii. Private clinic iv. Health center, and v. Self-referral [3]. The major diagnosis category was classified into i. Traumatic ii. Non traumatic and presence of previous chronic illness [14]. The outcome (adult early mortality in ED) is divided into i. Very early ED mortality (is the occurrence of death within 24 h of arrival to the ED) ii. Death > 24–72 h presentation to ED iii. Death on arrival (death upon arrival to ED) [3, 4]. Based on the severity of illness or injury and the care required and or the target time, a triage system is categorized in a color code i. Red (Immediate treatment, emergency resuscitation needed) ii. Orange (need very urgent management and care within 10 min of presentation) iii. Yellow (refer to majors for urgent management within less than 1 h) iv. Green (refer to the designated area for non-urgent cases within less than 4 h) v. Black (refer to a doctor for certification in less than 2 h) [20]. At last, the cause of mortality was grouped as i. Respirator ii. Cardiac iii. Multi-organ failure iv. Cardio respiratory arrest V. Other [3, 5, 21, 22].

### Data processing and analysis

Data manually checked and entered into Epidata version 4.2.6 and transferred to Statistical Package for Social Science (spss) version 23 for analysis. Summary statistics, descriptive, frequency, mean, and proportion were determined for all variables. Subsequently, allpatients dead on ED arrival were excluded from further analysis to differentiate the group ofpatients who may have benefitted from early ED care. Time from ED presentation to death was then dichotomized into < 24 h and > 24–72 h. This < 24 h group is named as very early ED mortalities; this group was chosen for analysis because the majority of them represent majorly ill and injured patients who might benefit from timely and appropriate ED interventions. A chi-squared test with binary logistic regression was used to test for the impact of the variables on the possibility of dying within 24 h of ED presentation. Bivariate logistic regression with a crude odds ratio *p*-value < 0.2 and multivariate logistic regression with adjusted odds ratios were conducted for excluding potential confounding factors between the variables. *P*-value < 0.05 was considered statistically significant. The odds ratio with a 95% confidence interval was analyzed to verify the strength of association.

The cause of ED mortality was listed out and merged into traumatic and non-traumatic causes.

### Ethical consideration

Ethical clearance letters were received from Addis Ababa University College of Health Science, Ethical Approval Board. A support letter was obtained from the Department of Emergency Medicine to the clinical director of TASTH. Verbal ethical permission was gained from the emergency department of TASTH and Card Room staff before undertaking data collection. Confidentiality was kept in each step. Given the retrospective and secondary nature of the data, Addis Ababa University College of Health Science, Ethical Approval Board waived the participants’ consent. **A**ll methods were carried out in accordance with relevant guidelines and regulations.

## Results

Over the 2 years study period, a total of 30,086 adult patients visited the ED; 846 (2.8%) death was registered in ED. The times from ED arrival to patient death were as follows: dead on arrival 65 (7.6%), 604 (71.4%) deaths occurred within 72 h alive on arrival to ED, among these, 506 (59.8%) charts qualified the criteria for analysis (24 h 274 (54%) and > 24–72 h 232 (46%)), while the remaining 98 (11.6%) charts were excluded because of incomplete records.

Table 1 shows the socio-demographic characteristics of the study participants. The age range was from 13 to 95 with a mean age of 43.5 ± 17.33 years. More death occurs in males (52.8%) than females, with male to female ratio of 1.2:1. The majority of the participants came from Addis Ababa region (41%) with more than half (51%) of participants were self-referral. More than three fourth of study participants (86%) had no previous ED visits within the last 30 days. Of all the deaths analyzed for this study, (92.7%) were due to non-traumatic causes and 26% had > 48–1 week duration of symptom. Of all the participants included in this study, 44.5% were under the red triage category and more than half 54% arrive at the hospital by taxi **(**Table 1**)**.

**Table 1:** Socio-demographic and clinical characteristics of patients who died within 72 h of ED presentation from March 2018 to 2020 TASTH Addis Ababa; Ethiopia

Variables		Length of stay in ED before death				Total sample (***N*** = 506)	
		0–24 h (***N*** = 274)		> 24–72 h (***N*** = 232)			
		N	%	N	%	N	%
Sex	Male	153	55.8	114	49	267	52.8
	Female	121	44.2	118	50.9	239	47.2
Age	13–24	42	15	34	14.7	76	15
	25–54	148	54	119	51.3	267	52.8
	55–64	44	16	40	17	84	16.6
	≥65	40	14.6	39	16.8	79	15.6
Residence	Addis Ababa	107	39	100	43	207	40.9
	Oromia	84	30.6	87	37.5	171	33.8
	Amhara	40	14.6	21	9	61	12
	Tigray	6	2.2	5	2.2	11	2.2
	Others	37	13.5	19	8.2	56	11
Source of referral	Public hospital	69	25.2	67	28.9	136	26.9
	Private hospital	3	1	9	3.9	12	2.4
	Private clinic	6	2.2	7	3	13	2.6
	Health center	7	2.6	5	2.2	12	2.3
	Self	156	56.9	102	44	258	51
	ROPD	30	10.9	40	17	70	13.8
	Others	3	1.1	2	0.9	6	1
Mode of transportation	Ambulance	63	23	46	20	109	21.5
	Taxi	135	49.3	138	59.5	273	54
	Public transport	8	2.9	4	1.7	12	2.3
	Private car	34	12.4	19	8.2	53	10.5
	Walk-in/ carried	34	12.4	25	10.8	59	11.7
Previous ED visit within last 30 days	no ED Visits	226	82.5	209	90.1	435	86
	1	42	15	21	9	63	12.4
	2	6	2.2	2	0.9	8	1.6
Major cause of death	Traumatic	20	7.3	17	7.3	37	7.3
	Non-traumatic	254	92.7	215	92.7	469	92.7
Triage category	Red	153	55.8	72	31	225	44.5
	Orange	78	28.5	104	45	182	36
	Yellow	29	10.6	38	16.3	67	13.2
	Green	14	5	18	7.7	32	6.4
Investigations	None	56	20.4	23	9.9	79	15.6
	Laboratory	167	61	144	62	311	61.5
	Imaging	15	5.5	16	6.9	31	6.1
	Laboratory and imaging	36	13	49	21	85	16.8
Duration of the symptom	< 4 h	32	11.7	17	7.3	49	9.7
	4–24 h	89	32.5	39	16.8	128	25.3
	25–48 h	40	14.6	41	17.7	81	16.
	> 48 h-1 week	60	21.9	74	31.9	134	26.5
	> 1 week	53	19.3	61	26.3	114	22.5
Time of death	Day time	145	52.9	119	51.3	264	52.2
	Night time	129	47.1	113	48.7	242	47.8

Note Others: residence: Dire Dawa, Afar, SNNPRs (South Nation Nationalities Peoples Republic)

source of referral: police hospitals, private hospital

### Cause of death

The major causes of death (469) were medical emergency disease: the principal top three causes were, shock 186 (36.7%), respiratory disease 152 (30%), and sepsis 85 (16.8%). The primary causes of traumatic death (37) were: road traffic accident 16 (3.2%), fall injuries 8 (1.6%), assault 5 (1%), and suicide 5 (1%). By and large more than half of the primary causes of death had co-morbidities with secondary causes of mortality majorly includes, cancer, cardiac disease, hypertension, hematologic malignancy, diabetes, and asthma.

Of all patients who died within 72 h of presentation to the emergency department (506), the primary immediate cause of death was cardiorespiratory arrest 189 (37.4%). Together they present findings that confirm cancer 176 (34.8%) as the leading co-morbid disease in early ED mortality **(**Table 2**).**

**Table 2:** Frequency distribution of cause of death early ED mortality from March 2018 to 2020 TASTH Addis Ababa; Ethiopia

Cause of death		Length of stay in ED before death				Total sample (***N*** = 506)	
		0-24 h (***N*** = 274)		> 24–72 h (***N*** = 232)			
		N	%	N	%	N	%
Traumatic cause of death	Road traffic accident	8	2.9	8	3.4	16	3.2
	Assault	1	0.4	4	1.7	5	1
	Fall injuries	6	2.2	2	0.9	8	1.6
	Gunshot	0	0	1	0.4	1	0.2
	Stab	1	0.4	1	0.4	2	0.4
	Suicide	3	1.1	2	0.9	5	1
	Burn	0	0	1	0.4	1	0.2
Sites of injury	Head injury	8	2.9	11	4.7	19	3.8
	Chest	2	0.7	1	0.4	3	0.6
	Spinal cord	6	2.2	4	1.7	10	2
	Polytrauma	1	0.4	1	0.4	2	0.4
	Others	2	0.7	1	0.4	3	0.6
Non-traumatic /medical cause of death	Respiratory disease	91	33.2	61	26.3	152	30
	Cardiovascular disease	50	18.2	34	14.7	84	16.6
	Renal disease	27	9.9	42	18.1	69	13.6
	Neurologic disease	42	15.3	38	16.4	80	15.8
	Liver disease	11	4	15	6.5	26	5.1
	DM and its complications	2	0.7	6	2.6	8	1.6
	Sepsis	46	16.8	39	16.8	85	16.8
	Septic shock	50	18.2	35	15.1	85	16.8
	Other types of shock	55	20.1	46	19.8	101	20
	Upper GI bleeding	20	7.3	13	5.6	33	6.5
	Hematologic malignancy	29	10.6	43	18.5	72	14.2
	Bleeding disorder	9	3.3	13	5.6	22	4.3
	Others	21	7.7	29	12.5	50	9.9
Co-morbid disease	Cancer	96	35	80	34.5	176	34.8
	Cardiac disease	40	14.6	30	12.9	70	13.8
	Hypertension	25	9.1	16	6.9	41	8.1
	Hematologic malignancy	14	5.1	5	2.2	19	3.8
	Diabetes Mellitus	10	3.6	7	3	17	3.4
	HIV/AIDS	8	2.9	18	7.8	26	5.1
	Asthma	3	1.1	7	3	10	2
	Others	17	6.2	37	15.9	54	10.7
Immediate cause of death	Respiratory failure	59	21.5	55	23.7	114	22.5
	Cardiac failure	42	15.3	38	16.4	80	15.8
	Multi-organ failure	45	16.4	46	19.8	91	18
	Cardio respiratory arrest	115	42	74	31.9	189	37.4
	Others	12	4.4	20	8.6	32	6.3

Others: sites of injury extremities and abdominal injury

: Co-morbid disease: CKD, CLD, epilepsy, seizure

**:** Non-traumatic cause of death: wound site infection, abdominal diseases, gastritis

: Immediate cause of death: massive haemorrhage, unknown causes

### Factors associated with very early ED mortality

Table 3 displays the output of crude and adjusted odds ratios following logistic regression. In bivariate logistic regression analysis, sex, residence, source of referral, duration of symptom, co-morbid diseases, and triage category fulfilled the criteria of *p*-values < 0.2 and transferred to multivariate logistic regression.

**Table 3:** Binary and multiple logistic analysis of factors associated with very early ED mortality from March 2018–2020, Addis Ababa, Ethiopia

Variables		Duration in ED					
		0-24 h	> 24-72 h	***P***-value	COR 95% CI	***P***-value	AOR 95% CI
Age	13–24	42	34	0.56	0.83 (0.44,1.56)		
	25–54	148	119	0.45	0.83 (0.5,1.36)		
	55–64	44	40	0.82	0.93 (0.50,1.72)		
	> 65	40	39	1	1		
Sex	Male	153	114	0.13	0.76 (0.49,1.19)*	0.29	0.80 (0.53,1.20)
	Female	121	118	1	1		
Residence	Addis Ababa	107	100	0.06	1.02 (0.99,3.37)*	0.01	2.78 (1.36,5.68)**
	Oromia	84	87	0.03	2.02 (1.08,3.78)*	0.001	3.23 (1.58,6.54)**
	Amhara	40	21	0.96	1.02 (0.48,2.2)	0.76	1.14 (0.48,2.70)
	Tigray	6	5	0.47	1.62 (0.44,6.01)	0.1	3.79 (0.78,18.34)
	Others	37	19	1	1	1	
Triage category	Red	153	72	0.003	0.32 (0.15,0.67)*	0.001	0.24 (0.10,0.55)**
	Orange	78	104	0.81	0.91 (0.43,1.96)	0.51	0.75 (0.33,1.74)
	Yellow	29	38	0.80	0.9 (0.38,2.11)	0.26	0.58 (0.22,1.50)
	Green	14	18	1	1		
Mode of transportation	Ambulance	63	46	0.98	0.99 (0.52,1.89)		
	Taxi	135	138	0.26	1.39 (0.79,2.45)		
	Public transport	8	4	0.56	0.68 (0.18,2.51)		
	Private car	34	19	0.48	0.76 (0.35,1.63)		
	Walk-in/carried	34	25	1	1		
Source of referral	Private hospital	3	9	0.18	4.5 (0.49,41.25)*	0.13	6.82 (0.55,84.04)
	Public hospital	69	67	0.69	1.46 (0.24,6.98)	0.33	2.89 (0.35,23.98)
	Private clinic	6	7	0.60	1.75 (0.22,14.22)	0.47	2.43 (0.22,26.41)
	Health center	7	5	0.95	1.07 (0.13,8.98)	0.73	1.54 (0.14,17.21)
	Self	156	102	0.98	0.98 (0.16,5.97)	0.55	1.91 (0.23,15.62)
	ROPD	30	40	0.46	2.0 (0.31,12.72)	0.36	2.71 (0.32,23.03)
	Others	3	2	1	1		
Previous ED visit with in last 30 days	No ED visits	226	209	0.22	2.77 (0.55,13.9)*	0.45	2.56 (0.46,14.2)
	1	42	21	0.64	1.50 (0.28,8.08)	0.97	1.43 (0.24,8.58)
	≥2	6	2	1	1		
Duration of symptoms	< 4 h	32	17	0.02	0.45 (0.22,0.89)	0.33	0.66 (0.29,1.50)
	4–24 h	89	39	0.00	0.35 (0.21,0.50)*	0.02	0.47 (0.26,0.87)**
	25–48 h	40	41	0.60	0.86 (0.49,1.52)	0.57	1.22 (0.62,2.42)
	> 48–1 week	60	74	0.9	1.34 (0.63,1.71)	0.51	1.21 (0.68,2.15)
	> 1 week	53	61	1	1		
Major cause of death	Traumatic	20	17	0.99	1.00 (0.51,1.97)		
	Non-traumatic	254	215	1	1		
Co-morbid disease	Hypertension	25	16	0.11	0.5(.0.21,1.18)*	0.83	1.09 (0.50,2.36)
	Diabetes mellitus	10	7	0.36	1.73 (0.53,5.62)		
	Cardiac disease	40	30	0.16	0.61 (0.30,1.22)*	0.66	0.84 (0.27,2.63)
	Cancer	96	80	0.65	0.89 (0.54,1.46)		
	Hematologic malignancy	14	5	0.07	0.29 (0.08,1.12)*	0.76	0.84 (0.27,2.63)
	HIV AIDS	8	18	0.08	2.91 (0.88,9.63)*	0.05	2.72 (1.01,7.30)**
	Asthma	3	7	0.30	2.09 (0.51,8.54)	0.80	0.72 (0.27,3.25)
	Others	17	37	1	1		
Time f death	Day time	145	119	0.72	1.07 (0.75,1.52)		
	Night time	129	113	1	1		

COR = *P < 0.25; AOR** = *P* < 0.05

In the multivariate logistic regression, residence Addis Ababa (*p*-value 0.01 AOR = 2.78, 95% CI: 1.36–5.68) and Oromia (*p*-value 0.001 AOR = 3.23, 95% CI: 1.58–6.54)), and co-morbid disease HIV AIDS (*p*-value 0.05 AOR = 2.72, 95% CI: 1.01–7.30) were positively associated with very early ED mortality. Whereas, triage category red (*p*-value 0.001 AOR = 0.24, 95% CI: 0.10–0.55) and duration of symptoms 4–24 h (*p*-value 0.02 AOR = 0.47, 95% CI: 0.26–0.87) were negatively associated with very early ED mortality than early mortality with *p*-value < 0.05 (Table 3).

## Discussion

This study has retrospectively characterized the prevalence and early all-cause of adult ED mortality rate at Tikur Anbesa Tertiary Specialized Hospital. The total number of patients visited the ED TASTH over 2 years was 30,086 with an early ED mortality rate of 506 (59.8%). The magnitude of adult ED mortality in this study was higher than a study conducted in South Nigeria (8.6%) [21], Southwest Nigeria (5.2%), Federal Ido Ekiti State, Nigeria (3.9%), Uganda (22.8%), Congo (12.3%), and Tanzania (0.74%) [5]. This variance might be due to emergency department health care settings, management standards, resource setting, and cause of death difference. Very early death < 24 h after the presentation to ED 274 (54%) was higher than study in the similar resource setting 37% [3]. A possible explanation for this might be a difference in a study period.

Our findings of shock and respiratory arrest as the most common non-traumatic (medical) causes of death in TASTH adult ED. This is inconsistent with the study conducted in South-South of Nigeria, which mentioned cardiovascular disease as a major cause of death [20], finding in southwest Nigeria, lists stroke [5], and study in Tanzania mentioned anemia, malaria, HIV, respiratory disease, and cardio-circulatory disease [22] as a major cause of ED mortality. As to the gender composition, the relatively middle age group death is higher than other age groups. This is consistent with a previous ED study in Nigeria [21] and Ethiopia [3].

Our findings of a road traffic accident (3.2%) followed by fall injuries 1.6% as the most common traumatic cause of death among adult ED patients. The trauma burden among clients in Ethiopia adult ED patients is in line with previously reported ED mortality studies in developing countries [21, 23–25].

The present study confirmed the findings of head injury as the single most common cause of death in TASTH adult ED mortality. This finding is directly in line with the findings in Tanzania [25] and Kenya [26].

For traumatic death, studies showed road traffic accidents as a major cause of death among developing countries. These is evidenced in South-south of Nigeria (80.3%) [21], Federal Medical Centre IdoEkiti (13%) [24], in Kenya (41.7%) [26], and Ethiopia [27].

Multiple regression analysis revealed that residence, triage category, duration of symptom, and co-morbid disease have a significant association with very early mortality (death within 24 h).

Those patients who reside in Addis Ababa were nearly three times to be on the list of very early ED mortality (*p*-value 0.01 AOR = 2.78, 95% CI: 1.36–5.68). Likewise, those from the Oromia region also three times high likely to be a very early ED mortality case with (*p*-value 0.001 AOR = 3.23, 95% CI: 1.58–6.54). As the distance from the living area increases the time for patients to reach to health facility also delays, this potentially leads to ED mortality.

In the present study surprisingly the odds of triage category red is 0.2 times less likely to cause very early ED mortality (*p*-value 0.001 AOR = 0.24, 95% CI: 0.1–0.55). Those patients in the triage category red are in severe injury and/or illness and need an immediate intervention but high potential for survival with treatment. Thus, this leads to conclude that some cases under triage category red were easily reversible & treatable conditions, and/or this group of clients admitted in ED TASTH had got immediate intervention as required.

The odds of having symptom duration 4–24 h was nearly 0.5 times less likely to end with very early ED mortality (*p*-value 0.02 AOR = 0.47, 95% CI: 0.26–0.87) than their counterparts. The possible explanation for this might be, unlike patients with symptom duration < 4 h, patients with duration of symptom 4–24 h could be less severely injured and these patients have a little more time to get extended ED care which can potentially save their lives.

The sole predictor of very early ED mortality identified in this study was a co-morbid disease, HIV AIDS. Those clients with known seropositive status for HIV AIDS are three times more likely to be dead very early in ED than their counterparts (*p*-value 0.04 AOR = 2.72, 95% CI: 1.01–7.30). It is a scientific fact that HIV AIDS is an immune compromising disease, a person with a compromised immune system cannot able to handle additional disease and /or trauma. Thus, they are highly likely to be dead very early as compared to patients with other co-morbid disease.

### Strength and limitation of the study

The primary strength of this research is it’s a 2 years retrospective study and all qualified data were included. This highly increases the quality of representativeness. In addition, the finding provides baseline statistical data for ED mortality in TASTH and Ethiopian hospitals as a whole. The analysis of predictors of very early ED mortality offers essential information for the development of health policy and intervention to minimize early ED mortality.

Since the study is a retrospective chart review, triage records were at times incomplete concerning the leading cause of death. Another limitation of this study was missed charts which potentially limit the result. Finally, this study was limited to the collection and analysis of data contained in HMIS data in TASTH.

## Conclusions

The mortality burden of road traffic accident and shock in TASTH is significant and magnitude of ED mortality differs between these groups. Early ED mortality rate was higher among males than females and the majority of patients with shock and respiratory disease die within 24 h of presentation to ED. As emergency mortality increase in Ethiopia, the possible impact of interventions intended at minimizing mortality among clients with co-morbid illness, clients who reside in Addis Ababa and Oromia region should be encouraged. Emphasis should also be given to duration of symptom and triage category.

## Data Availability

The datasets used and/or analyzed during the current study are available from the first author on reasonable request.

## Abbreviations

ED: Emergency Department
LMICs: Low and Middle-Income Countries
TASTH: Tikur Anbesa Specialized Tertiary Hospital
ROPD: Regular outpatient department
MOF: Multi-Organ Failure
